# Development of a Prediction Model for Stress Fracture During an Intensive Physical Training Program: The Royal Marines Commandos

**DOI:** 10.1177/2325967117716381

**Published:** 2017-07-25

**Authors:** Maria T. Sanchez-Santos, Trish Davey, Kirsten M. Leyland, Adrian J. Allsopp, Susan A. Lanham-New, Andrew Judge, Nigel K. Arden, Joanne L. Fallowfield

**Affiliations:** † NIHR Musculoskeletal Biomedical Research Unit, University of Oxford, Botnar Institute of Musculoskeletal Sciences, Oxford, UK.; ‡ Arthritis Research UK Sports, Exercise and Osteoarthritis Centre, University of Oxford, Botnar Institute of Musculoskeletal Sciences, Oxford, UK.; § Environmental Medicine and Sciences Division, Institute of Naval Medicine, Hampshire, UK.; ‖ Nutritional Sciences Department, School of Biosciences and Medicine, Faculty of Health and Medical Sciences, University of Surrey, Surrey, UK.; ¶ MRC Lifecourse Epidemiology Unit, University of Southampton, Southampton General Hospital, Southampton, UK.; *Investigation performed at the Commando Training Centre, Lympstone, Devon, UK*

**Keywords:** stress fracture, risk factors, prediction, military, Royal Marines

## Abstract

**Background:**

Stress fractures (SFs) are one of the more severe overuse injuries in military training, and therefore, knowledge of potential risk factors is needed to assist in developing mitigating strategies.

**Purpose:**

To develop a prediction model for risk of SF in Royal Marines (RM) recruits during an arduous military training program.

**Study Design:**

Case-control study; Level of evidence, 3.

**Methods:**

RM recruits (N = 1082; age range, 16-33 years) who enrolled between September 2009 and July 2010 were prospectively followed through the 32-week RM training program. SF diagnosis was confirmed from a positive radiograph or magnetic resonance imaging scan. Potential risk factors assessed at week 1 included recruit characteristics, anthropometric assessment, dietary supplement use, lifestyle habits, fitness assessment, blood samples, 25(OH)D, bone strength as measured by heel broadband ultrasound attention, history of physical activity, and previous and current food intake. A logistic least absolute shrinkage and selection operator (LASSO) regression with 10-fold cross-validation was used to select potential predictors among 47 candidate variables. Model performance was assessed using measures of discrimination (c-index) and calibration. Bootstrapping was used for internal validation of the developed model and to quantify optimism.

**Results:**

A total of 86 (8%) volunteer recruits presented at least 1 SF during training. Twelve variables were identified as the most important risk factors of SF. Variables strongly associated with SF were age, body weight, pretraining weightbearing exercise, pretraining cycling, and childhood intake of milk and milk products. The c-index for the prediction model, which represents the model performance in future volunteers, was 0.73 (optimism-corrected c-index, 0.68). Although 25(OH)D and VO_2_max had only a borderline statistically significant association with SF, the inclusion of these factors improved the performance of the model.

**Conclusion:**

These findings will assist in identifying recruits at greater risk of SF during training and will support interventions to mitigate this injury risk. However, external validation of the model is still required.

The 32-week Royal Marines (RM) recruit training is one of the most arduous and longest initial military training programs in the world. Recruits are at relatively high risk of musculoskeletal overuse injuries (30%), including stress fracture (SF), which represents one of the more severe overuse injuries in military training.^42^

SF is a partial or complete fracture of bone, and it occurs when bones are repetitively loaded with vigorous weightbearing (WB) exercise over short time periods without sufficient time for repair. Existing research in military and athletic populations have documented that the incidence of SF ranges from 0.7% to 31%.^14,17,24^ The most prevalent site of SF is generally the tibia, followed by the metatarsal bones.^18^ Furthermore, it is the single most common cause for lost training days, and it represents a significant cost in terms of medical support and rehabilitation time as well as increasing the likelihood of recruits leaving training prior to completion.

The etiology of SF is multifactorial, and knowledge of potential risk factors is required to assist in developing mitigating strategies. Previous prospective studies in military populations have assessed SF risk factors in the Indian Army,^8^ US military,^1,27,28,32,47,48^ Israeli military,^14,15,33^ and Finnish Army,^31,55^ all of which have training programs that range between 8 and 16 weeks. However, there are only a few data assessing risk factors during longer training programs such as RM training.^9,10,36^ Previous systematic reviews^18,37,57^ have identified the following SF risk factors: older age, female sex, lifestyle habits (smoking and alcohol ingestion), low bone mineral density (BMD), previous lower limb injury, and poor nutrition. Because these variables are of limited predictive value when considered in isolation, the combination of them is needed for better predictive accuracy.

The problem of selecting a set of potential risk factors to include in regression modeling is well known, but it is also among the most controversial and difficult tasks in epidemiologic analysis.^56^ Selecting a practical number of predictors to be included in the model is the natural first step, and they are generally selected based on subject knowledge from clinical expertise and reviews of the literature.^23,50,51^ Currently, there is a lack of research on the risk factors for SF in an elite military setting; therefore, we used a more statistically driven method of predictor selection. For this study, given the sample size and incidence of outcome, standard variable selection methods (forward selection and/or backward elimination) could lead to only a limited number of predictors being considered to avoid model overfitting. An alternative method that overcomes this limitation is LASSO (least absolute shrinkage and selection operator) regression and is the most widely used.^38,49^ LASSO is a powerful penalized regression method used in predictor selection.^34^

The purpose of this study was to identify the most probable SF risk factors in RM recruits at the start of military training and to construct an SF prediction model using advanced statistical methods. The resulting better understanding of the interrelationship between SF risk factors will assist in developing evidence-based preventive interventions and safety promotion programs for mitigating SF in the military.

## Methods

In this study, we followed the TRIPOD (transparent reporting of a multivariable prediction model for individual prognosis or diagnosis) statement^6^ to report the prediction model, including model development, model performance, and model internal validation.

### Study Population

The present study used data from phase II of Surgeon General’s Bone Health Project (SGBHP). SGBHP adopted a prospective, observational study design to assess the relationship between nutritional influences on bone health and SF occurrence during the 32-week recruit training program at the Commando Training Centre Royal Marines (CTCRM), Lympstone, Devon, UK. The RM is an all-male elite fighting force; therefore, there were no females in the sample. Recruits who successfully completed the physical and professional selection tests, and who were deemed medically fit and healthy to undertake RM training, were eligible to participate in the study.

A total of 1113 recruits from 20 troops of RM recruits, commencing training between September 2009 and July 2010, were invited to participate in this study. Written informed consent was obtained from volunteer recruits (n = 1090; 98% response rate) (age range 16-33 years); 23 recruits declined to participate in the study. A further 8 recruits were discharged during the first week due to preexisting medical conditions, leaving 1082 participants.

### Ethical Considerations

The study was approved by the UK Ministry of Defence Research Ethics Committee and was conducted in accordance with the ethical standards of the Declaration of Helsinki.

### Primary Outcome: Stress Fracture

Recruits reporting to the CTCRM Medical Centre with symptoms of a potential SF underwent examination and radiography or magnetic resonance imaging (MRI) scanning to confirm SF diagnosis as part of routine care. SF diagnosis was based on a positive radiograph or MRI scan. Depending on the fracture site, a negative initial radiograph was followed up by a further radiograph or MRI to confirm diagnosis. All recruits with SF were removed from RM training and underwent rest and rehabilitation in situ under medical supervision.

### Potential Risk Factors

Based on the information available in the study and on previous research in the scientific literature, 47 potential predictors from measurements collected at the start training were selected to be included in the modeling analyses; categories included physical fitness, diet, lifestyle, education, season for commencing RM training, and measures of bone health.

### Recruit Information

Age, education, and the season of the year at the start of training were available. As the association of age on SF was nonlinear, it was divided into 3 categories based on distributions from a previous study associated with discharge in US Marine Corps recruits^40^: younger than 19 years (range, 16-18 years), 19 to 23 years, and 24 years or older (range, 24-32 years). Education was defined as secondary school versus further education/degree, and season when the recruit started training was divided into the 4 standard seasons (autumn, winter, spring, and summer).

### Anthropometric Assessments

Height and body weight were measured. Height was measured in centimeters (to the nearest 0.1 cm) in a standing position, with shoes/boots removed, on a stadiometer (Invicta), with feet together. Body weight was measured in kilograms (Seca) in standard-issue t-shirt and shorts.

### Self-Reported Dietary Supplement Usage

Multivitamins and minerals, creatine, sports/energy drinks/energy gels, and protein bars/powder/shakes were included in an assessment of self-reported pre-RM training dietary supplement usage. Categorical variables of intake frequency for each dietary supplement were generated (*never*, *sometimes*, and *every day*).

### Lifestyle Habits

Smoking habits and alcohol intake were assessed. Recruits were classified as never smokers, ex-smokers, or current smokers. Alcohol intake was considered relative to intake in units per week, and it was used as a continuous variable.

### Broadband Ultrasound Attenuation Measurement

Broadband ultrasound attenuation (BUA) measurement was assessed on the dominant and nondominant foot (dB·MHz^−1^) as an indicator of bone strength.^26^ It is considered to be a rapid, safe, and relatively inexpensive technique for measuring skeletal status.^20^ This measure was taken across the calcaneum of a seated recruit. A continuous score was used for the analysis, where a greater BUA was indicative of higher bone mass and greater bone strength.

### Royal Marine Fitness Assessment

The Royal Marine Fitness Assessment (RMFA) is composed of 4 parts, which include the Multistage Fitness Test (MSFT)^3^ to estimate maximum oxygen uptake (VO_2_max),^29^ a push-up test, a sit-up test, and a pull-up test. The 4 fitness tests were undertaken in a gymnasium with recruits wearing shorts, t-shirt, and training shoes. The recruits were required to do the maximum number of push-ups, sit-ups, and pull-ups in 60 seconds. All measures were treated as continuous for the analysis.

### Exercise Pre-RM Training Preparation

Mode, duration, frequency, and volume of exercise pre-RM training preparation were included. Mode of training was assessed by the amount of WB and non-WB exercises from a list of 3 WB exercises (ie, running, circuit training, and weight training) and 2 non-WB exercises (ie, cycling and swimming). Duration was assessed by the number of weeks of pre-RM training preparation, frequency by the number of training sessions per week, and volume by the minutes per week training.

Previous lower-limb injuries (dominant and nondominant leg) were also self-reported by recruits.

### Assessment of Micronutrient and Vitamin D Status

A nonfasting blood sample was drawn by medical personnel, using serum separation vacutainers. Serum samples were provided for magnesium (as marker of micromineral status), zinc, selenium, copper (as markers of trace element status), and serum 25(OH)D concentration (as marker of vitamin D status). A threshold of 50 nmol/L for 25(OH)D was used for the analysis.^10^

### Physical Activity and Dietary Intake Measurements

A validated survey, the Food Frequency Questionnaire,^11,35^ which examines childhood, adolescence, and current diet and physical activity levels, was administered to recruits at the beginning of RM training.

Dietary intake focused on recruit eating choices just prior to commencing RM training (the past month), as well as during childhood and adolescence, as an assessment of habitual dietary patterns. Milk, milk products (such as yogurt, cream, ice cream, custard, milk puddings), vegetables, and fruit were included. Intake of each group was determined in times/week (except vegetables and fruit, which were determined in portions/week).

Current activity levels were assessed by number of minutes walked per day, number of minutes cycled per day,^39^ and by the following 2 questions: “During your working time and during your nonworking time, how often during a normal week were you physically active for at least 20 minutes during which time you became short of breath and sweat?” Recruits were classified into 3 groups: once or less per week, 2 to 3 times per week, and more than 3 times per week. Physical activity throughout childhood and early adulthood (ie, 0-12 years and 12-18 years) was assessed by asking recruits how often they were normally physically active for at least 20 minutes during which time they became short of breath and sweaty: Once or less per week, 2 to 6 times per week, more than 6 times per week.

Table 1 provides a detailed description of all covariates recorded.

**TABLE 1:** List of Prognostic Variables for Stress Fractures Available for Analysis*^a^*

Variable	Additional Information	Variable	Additional Information
Age	(1) 16-18 y; (2) 19-23 y; (3) 24-32 y	RMFA	
Education	(1) Secondary school; (2) Further education/degree	VO_2_max	mL·kg^−1^·min^−1^ (continuous)
Season at start of training	(1) Autumn; (2) Winter; (3) Spring; (4) Summer	Push-ups	Counts (continuous)
Anthropometric assessment		Sit-up tests	Counts (continuous)
Height	Meters (continuous)	Pull-up test	Counts (continuous)
Body weight	Kilograms (continuous)	Exercise pre-RM training	
Dietary supplement usage self-reported		Amount of WB exercises	(1) 0-2 WB exercises; (2) 3 WB exercises
Multivitamins with minerals	(1) Never; (2) Sometimes; (3) Every day	Amount of non-WB exercises	(1) 0-1 non-WB exercises; (2) 2 non-WB exercises
Creatine	(1) Never; (2) Sometimes; (3) Every day	Duration	Number of weeks (continuous)
Sports/energy drinks/ energy gels	(1) Never; (2) Sometimes; (3) Every day	Frequency/wk	Times per week (continuous)
Protein bars/powder/shakes	(1) Never; (2) Sometimes; (3) Every day	Weekly training volume	Minutes (continuous)
Lifestyle habits		Previous injury	
Smoke	(1) Never; (2) Ex-smoker; (3) Current	Lower limb injury in dominant leg	Yes or No
Alcohol	Units/wk (continuous)	Lower limb injury in nondominant leg	Yes or No
BUA			
Dominant foot	dB·MHz^−1^ (continuous)		
Nondominant foot	dB·MHz^−1^ (continuous)		
Blood sample		Dietary intake FFQ	
Magnesium	µmol/L (continuous)	Current	
Zinc	µmol/L (continuous)	Milk intake	(1) Low (<285 mL/d); (2) Moderate (426 mL/d); (3) High (≥852 mL/d)
Selenium	µmol/L (continuous)	Milk products intake (eg, yogurt and cream)	(1) Low (<2 times per week); (2) Moderate (2-5 times per week); (3) High (≥5 times per week)
Copper	µmol/L (continuous)	Fruit intake	(1) Low (<8 portions per week); (2) Moderate (8-13 portions per week); (3) High (≥14 portions per week)
25(OH)D	<50 nmol/L vs ≥50 nmol/L	Vegetable intake	(1) Low (<14 portions per week); (2) Moderate (14-22 portions per week); (3) High (≥23 portions per week)
FFQ: Physical activity		Adolescence and childhood	
Current		Milk intake	(1) Low (<285 mL/d); (2) Moderate (426 mL/d); (3) High (≥852 mL/d)
Minutes/d of walking	(1) <30 min/d; (2) ≥30 min/d	Milk products intake (eg, yogurt, cream, ice cream, custard, milk puddings)	(1) Low (<4 times per week); (2) Moderate (4-5 times per week); (3) High (≥6 times per week)
Minutes/d of cycling	(1) <30 min/d; (2) ≥30 min/d	Fruit intake	(1) Low (<4 times per week); (2) Moderate (4-5 times per week); (3) High (≥6 times per week)
Physical activity during working time	(1) Once or less/wk; (2) 2-3 times/wk; (3) >3 times/wk	Vegetable intake	(1) Low (<4 times/wk); (2) Moderate (4-5 times/wk); (3) High (≥6 times/wk)
Physical activity during nonworking time	(1) Once or less/wk; (2) 2-3 times/wk; (3) >3 times/wk		
Adolescence and childhood			
Physical activity	(1) Once or less/wk; (2) 2-6 times/wk; (3) >6 times/wk		

*^a^*BUA, broadband ultrasound attenuation measurement; FFQ, Food Frequency Questionnaire; RM, Royal Marines; RMFA, Royal Marine Fitness Assessment; VO_2_max, maximum oxygen uptake; WB, weightbearing.

### Statistical Analyses

Descriptive statistics of all potential predictors, according to whether the recruit presented or did not present an SF during RM training, were examined using means (SD) or medians (interquartile range) for quantitative measures, and frequency (percentage) for categorical variables.

Linearity assumption for continuous variables (using fractional polynomials or linear splines) was assessed, and the presence of interactions between age and the other variables was tested.^43^ To fill in variables with missing values, and because there was less than 15% missing data (see Appendix Table A2), a stochastic simple imputation method was used. It was created as the first of a series of 10 multiple imputations using MICE (multiple imputation by chained equation)^45^ (see Appendix Table A1). All prespecified predictors were included in the imputation model, together with the outcome.

The predicting model was achieved in 2 steps. First, LASSO shrinkage logistic regression method^54^ was used to reduce the final model to the most important variables to predict SF. It shrinks the coefficient estimates toward zero, with the degree of shrinkage depending on an additional parameter, lambda (λ) (this study used λ = 1). A single model adjusted for all potential variables was fitted with a 10-fold cross-validation and the minimum average mean-squared error (MSE) to extract the nonzero coefficients and therefore the significant predictors. This method focuses on the overall fit (best model fit) rather than statistical significance of individual predictors. As a consequence, predictors with a *P* > .05 could still be included in the final model. Second, odds ratios (ORs) and 95% confidence intervals (CIs) using a classic logistic regression model, were estimated for the principal risk factors selected in the previous step.

### Internal Validity

To check the internal validity of the model, 200 bootstrap samples with replacement were used to assess bias-corrected estimates of predictive ability.^22^ The evaluation of the model performance considered measures of discrimination and calibration.^5,44^ Discrimination was assessed using the c-index (this value varies between 0 and 1, where 1 represents perfect discrimination).^21^ In logistic regression, c-index is identical to the area under the receiver operating characteristic (ROC) curve. Calibration was assessed by calibration plots.

All calculations were performed using Stata statistical software version 13.1 (StataCorp) and R statistical software, version 3.2.3 (R Foundation for Statistical Computing). Variable selection and internal validation of the model were performed using the “glmnet”^19^ and the “rms”^16^ packages, respectively.

## Results

### Recruit Characteristics

**Figure 1.**
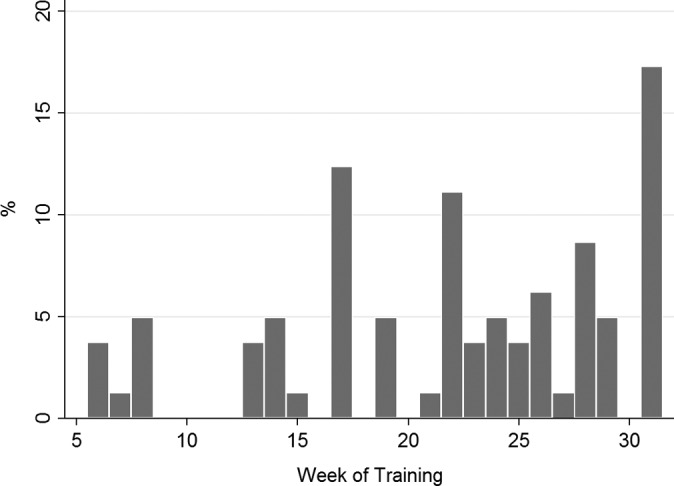
Distribution (%) of first stress fracture in 1082 recruits during 32-week training.

During phase II of SGBHP, a total of 86 recruits (8% of the study cohort) suffered at least 1 SF during the 32-week training period, with the metatarsal as the most common injury site (44 recruits), followed by the tibia and fibula (34 recruits). The majority of SFs (∼80%) occurred in the latter 15 weeks of RM training (Figure 1). The highest frequency was in week 31 (17.3%), followed by week 17 (12.4%) and week 22 (11.1%).

The proportion of missing data for each variable included in this study is shown in Appendix Table A2. The missing data represented less than or equal to 10% for all variables.

Distributions of the all-potential predictors by SF status are presented in Appendix Table A1. Significant differences (*P* < .05) between recruits who sustained an SF and those who did not were found for age, body weight, BUA of the dominant foot, volume of WB exercise pre-RM training, minutes per day of cycling, and milk intake during childhood. Compared with the recruits without SF, recruits with at least 1 SF during training were older, had a lower body weight, and had lower units of BUA of the dominant foot. In addition, recruits who reported a high amount of WB exercise pre-RM training preparation (3 WB exercises), cycling 30 minutes or more per day, and low intake of milk during their childhood were more likely to incur an SF.

### Selection of Principal Predictors of Stress Fracture

Twelve variables were selected by the LASSO selection approach. The model contained age, body weight, smoking habit, BUA of the dominant foot, VO_2_max, 25(OH)D concentration, amount of WB exercise, minutes cycling per day, physical activity during childhood, vegetables intake during adolescence, and milk and milk products intake during childhood.

The ORs and 95% CI of the predictors are presented in Table 2. Higher SF risk was associated with older age group (range, 24-32 years) (OR, 1.98; 95% CI, 1.07-3.55) compared with recruits between 19 and 23 years old. Increased WB exercise pre-RM training (OR, 2.46; 95% CI, 1.51-4.00), high frequency of cycling per day pre-RM training preparation (OR, 1.71; 95% CI, 1.07-2.74), and high intake of milk products during childhood (OR, 1.84; 95% CI, 1.03-3.30) were also associated with increased risk of SF.

**TABLE 2:** Estimation for Principal Risk Factors of Stress Fracture During Royal Marine Recruit Training*^a^*

Predictor Variable (Reference Category)	OR	95% CI	*P* Value*^b^*
Age, y (19-23)			
<19	1.66	0.97-2.85	.066
>23	1.98	1.07-3.55	**.030**
Body weight, kg	0.96	0.93-0.99	**.018**
Smoke (never)			
Ex-smoker	1.55	0.91-2.64	.109
Current	0.73	0.32-1.64	.447
BUA of the dominant foot, dB·MHz^−1^	0.99	0.98-1.00	.150
VO_2_max, mL·kg^−1^·min^−1^	0.93	0.86-1.01	.074
Amount of WB exercise (0-2 exercises)			
3 exercises	2.46	1.51-4.00	**<.001**
25(OH)D, nmol/L (≥50)			
<50	1.56	0.95-2.56	.077
Minutes/d of cycling (<30)			
≥30	1.71	1.07-2.74	**.026**
Physical activity in childhood (once or less/wk)			
2-6 times/wk	1.27	0.54-2.96	.584
>6 times/wk	1.76	0.79-3.93	.165
Adolescence			
Vegetables, times/wk (Low, <4)			
Moderate (4-5)	0.67	0.36-1.24	.200
High (≥6)	0.91	0.51-1.60	.735
Childhood			
Milk intake, mL/d (Low, <285)			
Moderate (426)	0.95	0.57-1.61	.862
High (≥852)	0.45	0.23-0.86	**.016**
Milk products, times/wk (Low, <4)			
Moderate (4-5)	1.19	0.67-2.11	.550
High (≥6)	1.84	1.03-3.30	**.039**
Model intercept	30.8		
c-index, fitted model (95% CI)	0.73 (0.67-0.78)		
Optimism	0.05		
Bias-corrected c-index	0.68		

*^a^*BUA, broadband ultrasound attenuation; WB, weightbearing.

*^b^*Boldfaced *P* values indicate statistical significance (*P* < .05).

In contrast, variables strongly associated with a lower risk of SF were high body weight (OR, 0.96; 95% CI, 0.93-0.99); and high milk intake during childhood (OR, 0.45; 95% CI, 0.23-0.86) compared with low intake. Variables with a borderline statistically significant association with SF included VO_2_max and 25(OH)D. Recruits with poor aerobic fitness at the start of training and low concentrations of 25(OH)D (<50 nmol/L) were associated with an increased risk of SF during training.

Smoking habits, bone strength of the dominant foot, physical activity during childhood, and vegetable intake during adolescence contributed to the overall model performance, although they did not have a statistically significant association with SF.

**Figure 2.**
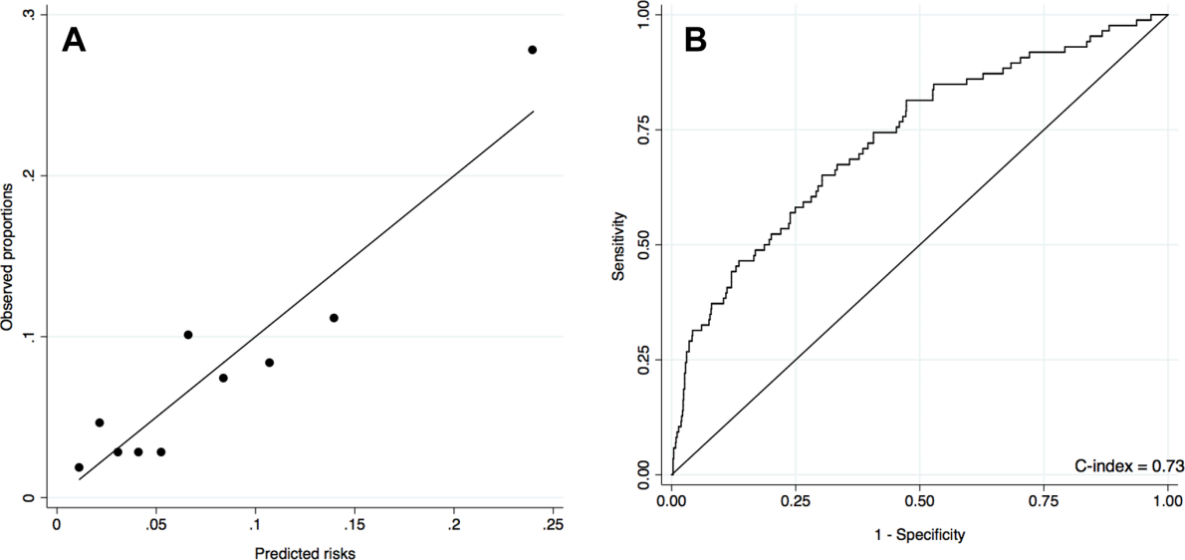
(A) Calibration curve of the prediction model of the risk of stress fracture during 32-week Marines training. The solid line indicates perfect calibration. (B) Receiver operating characteristic curve plot to assess discrimination of the predictive model.

The performance of the model showed adequate calibration (Figure 2A) and discrimination (Figure 2B), with a c-index of 0.73 (95% CI, 0.67-0.78). Using bootstrap validation, the optimism-corrected c-index was 0.68, which indicated a moderate predictive model in future volunteers.

## Discussion

This study identified pretraining predictors of developing SF during the 32 weeks of RM recruit training, by using advanced statistical methods.

Age, body weight, exercise pre-RM training, and childhood milk intake were the strongest predictors of SF in the model. Although 25(OH)D, VO_2_max, smoking habits, bone strength of the dominant foot, physical activity during childhood, and vegetables intake during adolescence were weakly associated with SF (*P* > .05), the inclusion of these factors improved the performance of the model; hence, the effects attributable to these factors were small but important to explain the outcome. The predictive model had a reasonable prediction capacity and validity to identify SF in RM recruits during the training period (see Table 2).

### What Do We Already Know?

The occurrence of SF during training in this population was 8%, and it was consistent with rates reported for other military recruit populations in Europe.^55^ Many of the variables identified as important risk factors have been shown to predict the risk of SF in earlier studies,^24,53^ supporting the plausibility of this model.

Age and body weight were significantly associated with SF. Older age (24-32 years) predicted SF in the present population, compared with recruits between 19 and 23 years old, which is in agreement with previous findings in other military and nonmilitary populations.^4,31^ Higher body weight was significantly associated with a decreased risk of SF, and this association has been well documented.^15,36^

Within this study, VO_2_max and 25(OH)D concentrations were borderline significantly associated with SF during training. Recruits with lower aerobic fitness assessed in week 1 of training were more likely to have an increased risk of SF during the subsequent 32 weeks of training. These finding were consistent with a previous report that used the Cooper test as a measure of physical fitness.^55^ However, a lack of association between VO_2_max and SF incidence has been reported in a study of Israeli infantry recruits.^52^ It should be noted that recruits in the present study were volunteers joining one of the United Kingdom’s elite Service Arms, where recruitment is partly based on having a high aerobic fitness at the start of training.

There is controversy with regard to 25(OH)D; previous studies found an association between low serum 25(OH)D levels and increased SF risk,^10^ whereas others have found no evidence of an association in military personnel.^7^ The present study found that low levels of 25(OH)D (<50 nmol/L) were associated with greater risk of SF during training, compared with recruits with higher levels of 25(OH)D, and this result was consistent with previous studies in this population.^10^

The present study aimed to produce a statistical model with the optimum predictive ability, and not to formally assess the association of 25(OH)D with SF per se. Thus, the scope of this present study, in developing an expansive prediction model, may explain the weak association for 25(OH)D.

### What Does This Study Add?

This study has produced the first risk prediction model for SF during elite military training, using a wide variety of risk factors and excellent follow-up data. This model had a reasonable predictive capacity and validity to identify occurrence of SF. After appropriate external validation, this model may be useful in helping to identify recruits as well as athletes at greater risk of SF, and hence may contribute to the development of strategies to mitigate SF risk.

Although there are several well-established risk factors for SF,^18,37,57^ a novel finding from this study was the identification of new exercise and diet variables as predictors of SF, using an advanced variable selection method.^54^ Increased WB exercise pretraining was a significant risk factor of SF occurrence. This result does not agree with the majority of previous basic military studies, which have reported that recruits who had a physically active lifestyle in the past would be less likely to suffer SF when starting a vigorous exercise program.^8,25,47^ Possible explanations for this may be the following: First, as most SFs of foot and ankle are caused by repetitive vigorous WB activities such as running and marching, and they usually occur when individuals change their activities (eg, such as trying a new exercise, increasing the intensity of their workout, or changing the workout surface), well-conditioned individuals who have been preparing to join the RM could suffer injuries during the military training. Second, differences could be due to collecting pre-RM previous training data. High frequency of cycling per day pre-RM training preparation was associated with increased risk of SF. Cycling is an aerobic, non-WB sport that has been associated with lower BMD.^46^ Since BMD has been found to be a predictor of high risk of SF,^9^ high intensity of cycling pre-RM training may contribute to the development of SF because of its influence on bone development.

An interesting finding in the present study was the association between SF and the intake of milk and milk products during childhood. The model shows that recruits who reported a high intake of milk had a lower risk of SF than recruits with low intake of milk. A possible explanation for this is that in growing children, long-term avoidance of milk is associated with smaller stature and poorer BMD.^41^ As mentioned above, BMD is associated with SF; hence, inadequate childhood calcium intake may affect SF development during training due to its influence on bone health. A significant skeletal growth phase at childhood age may be particularly important to the prevention of SF.

The opposite relationship was found for milk products intake. High intake of milk products (yogurt, cream, ice cream, custard, milk puddings, etc) during childhood was statistically associated with increased risk of SF compared with a low intake. Milk products, in this study, could be related to fat products, so a high-fat dietary pattern could be associated with greater risk of SF, as inadequate nutritional intake may alter bone metabolism and predispose toward appearance of SF. Another possible explanation for this would be the lack of precision on reporting milk products.

To our knowledge, this is the first study to report an association between past dietary intake and risk of SF during male military training.

### Strengths and Potential Limitations

This study has a number of strengths, including unique prospective data, a wide range of potential risk factors, and low proportion of missing data. The study used a rigorous and robust variable selection method to reduce the number of potential risk factors for SF. For prognostic studies, the LASSO regression could select the most important variables much more efficiently than the standard variable selection methods, by omitting additional and redundant variables.^34^ LASSO controls multicollinearity and is also applicable in settings where the number of variables is higher than the sample size, where traditional logistic regression would fail.^54^ Twelve factors were identified to be included into the final model, but 8 factors were identified as the most important risk factors of SF occurrence during RM training. Established measures of prediction performance, including the overall model fit, discrimination, and calibration, suggested that the final model had a satisfactory performance. A further strength of this study was the use of the RM data set, where there had been very high recruitment and retention rates.

There are several potential limitations to this study. First, because we used the LASSO methods, which variables are “clinically important” could not be defined because if 2 predictors were perfectly collinear, the LASSO will pick one of them essentially at random. Second, there are currently no common methods to incorporate multiple imputation with LASSO; therefore, single imputation was used. Single imputation may underestimate associations, and point estimates are potentially unstable, although the low level of missingness (<10%) in the data combined with the size of the data set make this unlikely.^49^ Third, although the inclusion of recruits who did not complete training for noninjury reasons (n = 465) in logistic regression analysis may introduce bias, excluding them would reduce the statistical power and validity of the study. An analysis excluding these recruits was performed, and no significant impact on the results and interpretation of this study was found. Fourth, the results of this study were restricted to male military personnel, which may not be generalizable to women or to a more general population. Future research should focus on the relative contribution of general population and sex-specific conditions. Fifth, residual bias may exist. The development of the model in the present study only took into account variables at the start of training. Other possible factors during training could explain the high variability in outcome. Sixth, self-reported past physical activity and diet is subject to the weakness of recall bias. However, past measures have been found to be positively correlated with those recorded objectively at the same time period,^2,12,13,30,58^ and hence the Food Frequency Questionnaire, used in this study, represents a valid instrument for assessing past physical activity and food intake. Seventh, an SF may have remained undiagnosed for several weeks and may even have remained unreported in some recruits, so SFs may have been underreported in this study. Finally, these results require validation through further prospective studies to improve the predictive capacity of the model. However, the results of the present study provide new important predictors of SF cases.

## Conclusion

This model has provided an important contribution to the prediction of SF during RM training, identifying high-risk recruits for targeted injury-prevention studies. SF risk during training may be modified through adjustments to selection. Information from this study could be used to determine recruits at risk of developing an SF. Further replication in additional data sets may lead to further enhancement of the current model for RM and other military training programs.

## Appendix

**TABLE A1:** Descriptive Statistics of Recruits at Week 1 According to Stress Fracture During Training (Complete Cases vs Simple Imputation)^a^

Variable	Complete Cases		*P* Value	Simple Imputation		*P* Value
	Stress Fracture	Non–Stress Fracture		Stress Fracture	Non–Stress Fracture	
Recruit characteristics						
Age, %			.012			.011
<19 y	36.1	27.2		36.1	27.4	
19-23 y	38.4	55.0		38.4	55.0	
>23 y	25.6	17.8		25.6	17.6	
Education, %			.168			.158
Secondary school	53.5	45.7		53.5	45.6	
Further education/degree	46.5	54.3		46.5	54.4	
Anthropometric assessment						
Height, m, mean (SD)	1.8 (0.1)	1.8 (0.1)	.524	1.8 (0.1)	1.8 (0.1)	.561
Body mass, kg, mean (SD)	72.8 (8.0)	74.9 (7.6)	.020	72.8 (8.0)	74.8 (7.6)	.021
Health behaviors						
Smoke, %			.078			.078
Never	59.3	65.9		59.3	65.9	
Ex-smoker	31.4	21.2		31.4	21.2	
Current	9.3	13.0		9.3	13.0	
Alcohol, units/wk, median, (IQR)	12 (4-25)	14 (2-28)	.544	12 (4-25)	14 (2-28)	.545
BUA, dB·MHz^−1^, mean (SD)						
Dominant foot	94.0 (17.1)	98.5 (18.5)	.030	94.0 (17.1)	98.5 (18.8)	.035
Nondominant foot	94.5 (19.3)	96.0 (18.5)	.474	94.5 (19.3)	96.0 (18.5)	.496
RMFA						
VO_2_max, mL·kg^−1^·min^−1^, median (IQR)	51.9 (50.5-54.6)	52.5 (50.8-55.1)	.104	51.9 (50.5-54.6)	52.5 (50.8-55.1)	.052
Press-up, counts, median (IQR)	45 (36-52)	45 (39-53)	.335	44.5 (36-52)	45 (39-53)	.367
Sit-up tests, counts, median (IQR)	69 (56-85)	69 (56-85)	.439	68 (56-85)	68 (55.5-85)	.476
Pull-up test, counts, median (IQR)	8 (7-10)	8 (6-10)	.204	8 (7-10)	8 (6-10)	.336
Exercise pre-RM training preparation						
Number of WB exercises, %			.001			.001
0-2 WB exercises	32.6	50.6		32.6	51.4	
3 WB exercises	67.4	49.4		67.4	48.6	
Number of non-WB exercises (%)			.801			.779
0-1 non-WB exercises	57.0	58.4		57.0	58.5	
2 non-WB exercises	43.0	41.6		43.0	41.5	
Duration, wk, median (IQR)	24 (12-50)	20 (10-40)	.067	24 (12-48)	20 (10-40)	.099
Frequency, times/wk, median (IQR)	5 (4-6)	5 (4-6)	.783	5 (4-6)	5 (4-6)	.748
Weekly training volume, min, median (IQR)	360 (270-540)	360 (270-600)	.274	360 (270-540)	360 (270-550)	.328
Blood sample						
Magnesium, µmol/L, mean (SD)	1.0 (0.1)	1.0 (0.1)	.465	1.0 (0.1)	1.0 (0.1)	.558
Zinc, µmol/L mean (SD)	12.3 (3.6)	12.5 (2.5)	.424	12.6 (2.2)	12.3 (2.1)	.250
Selenium, µmol/L, mean (SD)	1.2 (0.1)	1.2 (0.2)	.182	1.2 (0.1)	1.2 (0.2)	.196
Copper, µmol/L, mean (SD)	14.0 (2.3)	14.0 (2.3)	.846	13.8 (2.3)	14.0 (2.3)	.485
25(OH)D, nmol/L, mean (SD)			.051			.067
<50 nmol/L, %	38.6	28.4		37.2	27.9	
≥50 nmol/L, %	61.5	71.6		62.8	72.1	
Physical activity FFQ						
Current						
Minutes/d of walking, %			.658			.672
<30 min	12.8	14.5		12.8	14.5	
≥30 min	87.2	85.5		87.2	85.5	
Minutes/d of cycling, %			.016			.012
<30 min	40.0	53.6		39.5	53.7	
≥30 min	60.0	46.4		60.5	46.3	
Physical activity during working time, %			.851			.807
Once or less/wk	29.1	26.5		29.1	26.0	
2-3 times/wk	12.8	12.4		12.8	12.6	
>3 times/wk	58.1	61.1		58.1	61.5	
Physical activity during nonworking time (%)			.497			.500
Once or less/wk	10.5	15.0		10.5	15.1	
2-3 times/wk	12.8	11.1		12.8	11.4	
>3 times/wk	76.7	74.0		76.7	73.6	
Adolescence						
Physical activity, %			.799			.800
Once or less/wk	7.1	8.6		7.0	8.8	
2-6 times/wk	27.4	29.5		27.9	28.9	
>6 times/wk	65.5	62.0		65.1	62.3	
Childhood						
Physical activity, %			.124			.224
Once or less/wk	9.5	15.6		9.3	12.9	
2-6 times/wk	28.6	37.1		30.2	36.2	
>6 times/wk	61.9	50.3		60.5	50.9	
Dietary intake FFQ						
Current						
Milk intake, mL/d, %			.974			.973
Low (<285)	16.3	16.7		16.3	16.7	
Moderate (426)	37.2	36.0		37.2	35.9	
High (≥852)	46.5	47.2		46.5	47.4	
Milk products, times/wk, tertiles			.386			.329
Low (<2)	20.9	27.8		20.9	28.4	
Moderate (2-5)	39.5	35.5		39.5	35.2	
High (≥5)	39.5	36.7		39.5	36.4	
Fruit, portions/wk, tertiles			.658			.518
Low (<8)	27.9	31.6		27.9	32.4	
Moderate (8-13)	30.2	31.2		30.2	31.6	
High (≥14)	41.9	37.2		41.9	35.9	
Vegetables, portions/wk, tertiles			.583			.527
Low (<14)	26.7	30.7		26.7	31.1	
Moderate (14-22)	40.7	35.4		40.7	34.9	
High (≥23)	32.6	34.0		32.6	33.9	
Adolescence						
Milk intake, mL/d, %			.572			.574
Low (<285)	12.8	15.0		12.8	14.9	
Moderate (426)	40.7	35.1		40.7	35.1	
High (≥852)	46.5	49.9		46.5	50.0	
Milk products, times/wk, %			.735			.721
Low (<3)	34.9	39.2		34.9	39.3	
Moderate (4-5)	33.7	31.2		33.7	30.9	
High (≥6)	31.4	29.6		31.4	29.8	
Fruit, times/wk, %			.876			.869
Low (<3)	25.6	23.3		25.6	23.2	
Moderate (4-5)	33.7	34.0		33.7	33.9	
High (≥6)	40.7	42.8		40.7	42.9	
Vegetables, times/wk, %			.347			.321
Low (<3)	29.1	25.3		29.1	24.7	
Moderate (4-5)	27.9	35.7		27.9	35.8	
High (≥6)	43.0	39.1		43.0	39.5	
Childhood						
Milk intake, mL/d, %			.077			.090
Low (<285)	38.4	31.1		38.4	31.3	
Moderate (426)	43.0	39.0		43.0	39.2	
High (≥852)	18.6	29.9		18.6	29.5	
Milk products, times/wk, %			.298			.303
Low (<3)	33.7	39.3		33.7	39.1	
Moderate (4-5)	31.4	33.6		31.4	33.7	
High (≥6)	34.9	27.2		34.9	27.3	
Fruit, times/wk, %			.507			.470
Low (<3)	38.4	35.6		38.4	34.9	
Moderate (4-5)	38.4	35.2		38.4	35.5	
High (≥6)	23.3	29.2		23.3	29.5	
Vegetables, times/wk, %			.769			.779
Low (<3)	31.4	35.1		31.4	35.0	
Moderate (4-5)	36.1	34.9		36.1	34.9	
High (≥6)	32.6	29.9		32.6	30.0	

*^a^*BUA, broadband ultrasound attenuation; FFQ, Food Frequency Questionnaire; IQR, interquartile range; RM, Royal Marines; RMFA, Royal Marine Fitness Assessment; WB, weightbearing.

**TABLE A2:** Missing Data

Variable	Missing, n (%)
Recruit characteristics	
Age, y	43 (4.0)
Education	58 (5.4)
Season at start training	—
Anthropometric assessment	
Height (m)	55 (5.1)
Body mass (kg)	55 (5.1)
Dietary supplement usage self-reported	
Multivitamins with minerals	—
Creatine	—
Sports/energy drinks/energy gels	—
Protein bars/powder/shakes	—
Health behaviors	
Smoke	46 (4.3)
Alcohol, units	57 (5.3)
BUA, dB·MHz^−1^	
Dominant foot	50 (4.6)
Nondominant foot	47 (4.3)
RMFA	
VO_2_max, mL·kg^−1^·min^−1^	111 (10.3)
Press-up test, counts	112 (10.4)
Sit-up test, counts	111 (10.3)
Pull-up test (counts)	112 (10.4)
Exercise pre-RM training preparation	
WB and non-WB exercises	59 (5.5)
Frequency/wk, times/wk	81 (7.5)
Duration, wk	105 (9.7)
Weekly training volume, min	93 (8.6)
Previous injury	
Lower limb injury in dominant leg	—
Lower limb injury in nondominant leg	—
Blood sample	
Magnesium, µmol/L	52 (4.8)
Zinc, µmol/L	57 (5.3)
Selenium, µmol/L	51 (4.7)
Copper, µmol/L	52 (4.8)
25(OH)D, nmol/L	69 (6.4)
Physical activity FFQ	
Current	
Walking, min/d	54 (5.0)
Cycling, min/d	61 (5.6)
Physical activity during working time	55 (5.1)
Physical activity during nonworking time	55 (5.1)
Adolescence	
Physical activity	62 (5.7)
Childhood	
Physical activity	61 (5.6)
Dietary intake FFQ	
Current	
Milk, mL/d	54 (5.0)
Milk products, times/wk	54 (5.0)
Fruit, times/wk	54 (5.0)
Vegetables, times/wk	54 (5.0)
Adolescence	
Milk, mL/d	54 (5.0)
Milk products, times/wk	54 (5.0)
Fruit, times/wk	54 (5.0)
Vegetables, times/wk	54 (5.0)
Childhood	
Milk, mL/d	54 (5.0)
Milk products, times/wk	54 (5.0)
Fruit, times/wk	54 (5.0)
Vegetables, times/wk	54 (5.0)

*^a^*BUA, broadband ultrasound attenuation; FFQ, Food Frequency Questionnaire; RMFA, Royal Marine Fitness Assessment; WB, weightbearing.
